# Rare Presentation of Pilar Cyst of the Thumb

**DOI:** 10.29252/wjps.8.2.259

**Published:** 2019-05

**Authors:** Kavish Maheshwari, Sandip Hindocha, Ali Yousif

**Affiliations:** Department of Plastic Surgery, Bedford Hospital NHS Trust, South Wing, MK429DJ, UK

**Keywords:** Pilar cyst, Trichilemmal cyst, Benign hand cyst

## Abstract

Pilar cysts are common cysts on the scalp and hair bearing area of the body. We found one such cyst on the dorsum of the thumb. There have been previous reports of them in the finger tips as a very rare occurrence. The site of this lesion supports the theory of a possible origin from the nail matrix. These lesions, even when found at unusual sites should have pilar cyst as a differential diagnosis. They must always be excised and subjected to careful histopathology to rule out proliferating trichilemmal cysts, which carry a rare risk of malignancy.

## INTRODUCTION

Cystic lesions of the skin are one of the most common lumps involving the skin and adnexal tissues.^1^ The most common term used is sebaceous cyst, which however now is acknowledged as two types of cysts including epidermal cysts, that arise from the epidermis and pilar cysts that arise from the piliary apparatus. Approximately 20% of epithelial cysts are trichilemmal cyst and 80% are epidermal.^2^ Pilar cysts were termed by Pinkus as trichilemmal cysts, when he identified the follicular isthmus of the external root sheath of the hair follicle to be giving rise to these.^3^^,^^4^ These lesions arise in areas of high follicular density with occasionally being found at back, vulva, nose, mons pubis, buttock, wrist, chest, elbow, or eyes.^5^^-^^8^ We present a case of pilar cyst in an extremely rare location.

## CASE REPORT

**Fig. 1:**
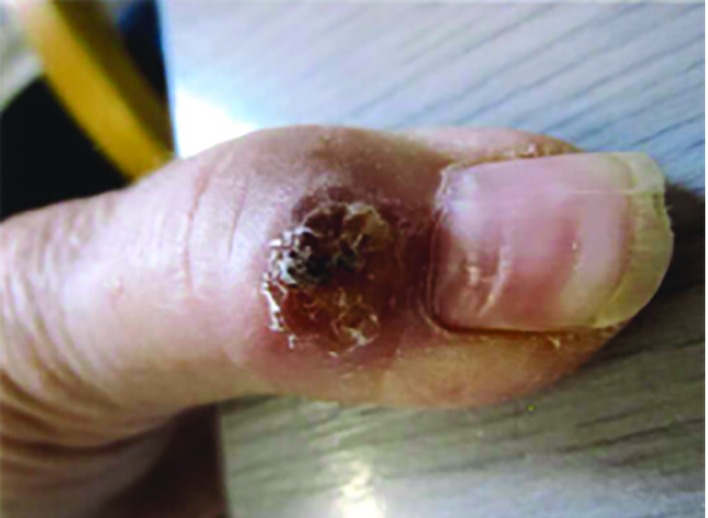
Appearance of lesion at presentation to the clinic

**Fig. 2:**
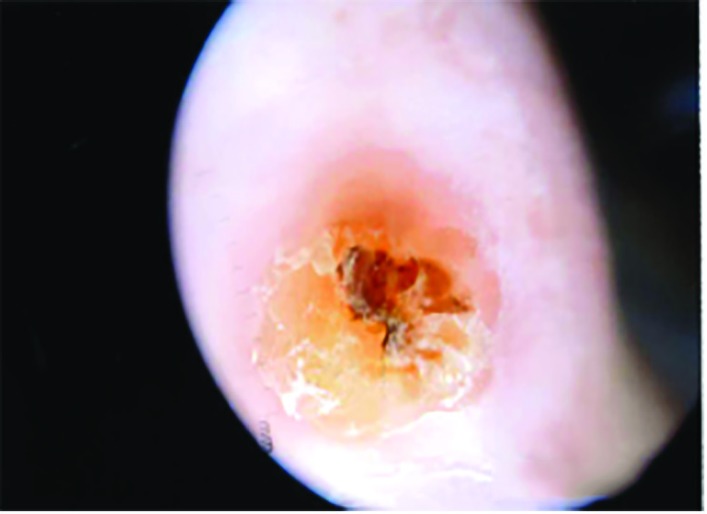
View through Dermatoscope

A 79-year old lady presented with a lump in her left thumb, which had been present for a few months. On examination, it was a small 1 by 1 cm lump proximal to the base of the nail, over the dorsal aspect of the distal phalanx of the left thumb. It had been slowly growing with no history of discharge or infection (Figure 1). She was referred as a possible differential diagnosis of mucoid cyst, epidermoid cysts or squamous cell carcinoma. An X-ray of the involved finger was also done, which did not show any bony spur (Figure 2). We did an excision of the lesion under local anesthesia. The procedure was uneventful and the lesion could be easily dissected from the surrounding tissue. The histopathology evaluation was suggestive of a cystic lesion lined by squamous epithelium and suggestive of an inflamed pilar cyst. There was no recurrence at her 3 month follow up and the operative site had healed well.

## DISCUSSION

Pilar cysts are usually a solitary intradermal or sub cutaneous lesion. They are clinically indistinguishable from epidermal cysts, but are far less common than them. They are most commonly found on the scalp.^3^^,^^9^ In contrast to epidermal cysts, they do not have a punctum. They can be easily enucleated. On gross examination, they are smooth walled cysts which on sectioning show cream white semi solid cheesy contents. They are lined by stratified squamous epithelium. Their keratinization is abrupt with no intervening granular layer and they contain homogeneous eosinophilic materials unlike the lamellated keratin flakes in an epidermal cyst.^3^

A previously held notion proposed that they could not arise in the palms and soles,^9^ however, it is no longer found to be true. There have been three reported cases of pilar cysts on the hand previously,^10^^-^^13^ but all of these lesions were present in the finger tips. They all arose in the non-hair bearing skin of the hand and in finger tips only. Our patient had it in the thumb and on the dorsum of the thumb, proximal to the nail bed. One of these case reports suggests the origin of this cyst as probably the nail matrix whose keratinization is trichilemmal.^12^ The location of this patient’s cyst seems to support this theory and offers an explanation to the origin of the cyst in a non-hair bearing part of the body.

Apart from the hand, they are also reported to arise in the anal region,^13^ penile region^14^ and around the eyes.^7^^,^^8^ The pathogenesis of these cysts in the perianal region was thought to develop on the wall of a previous hair follicle cyst as a result of trauma or inflammation. There is a poorly established suspected causal relationship with infection by the human papilloma virus.^13^ Interestingly, the trichilemmal cyst reported on the penis region occurred after a hypospadias repair. They hypothesized that the distal hypospadias repair had triggered squamous metaplasia with keratinization, which lead to the development of a trichilemmal cyst in a non-hair-bearing area of the body.^14^ The trichilemmal cyst identified in the eyelid, was also found at a site of previous chalazion excision.^8^ Thus, trauma seems to be the most likely cause of such cysts in unusual places.

These cysts may grow more extensively and form proliferating trichilemmal tumors, also called proliferating trichilemmal cysts, which are benign but may grow aggressively at the cyst site.^2^^,^^3^^,^^8^^,^^15^ Very rarely, trichilemmal cysts and proliferating trichilemmal tumors can undergo malignant transformation.^2^^,^^16^ The important differential diagnoses for a pilar cyst are proliferating trichilemmal tumors, proliferating epidermoid or infundibular cyst and trichilemmal carcinoma^3^ and thus warrant a removal always. We were referred this patient as a possible squamous cell carcinoma and we operated with a differential diagnosis of a mucoid cyst. Pilar cysts can arise in unusual locations of the body, even where they are devoid of any hair follicles. They are mostly preceded by some trauma, which may not always be documented. The need to rule out the possibility of proliferating pilar tumors is essential, since they have a rare risk of malignant transformation.

## CONFLICT OF INTEREST

The authors declare no conflict of interest.
